# Pentamidine inhibits prostate cancer progression via selectively inducing mitochondrial DNA depletion and dysfunction

**DOI:** 10.1111/cpr.12718

**Published:** 2019-11-13

**Authors:** Lin Liu, Fan Wang, Yu Tong, Lin‐Feng Li, Yanfeng Liu, Wei‐Qiang Gao

**Affiliations:** ^1^ State Key Laboratory of Oncogenes and Related Genes Renji‐Med‐X Clinical Stem Cell Research Center Ren Ji Hospital School of Medicine Shanghai Jiao Tong University Shanghai China; ^2^ School of Biomedical Engineering & Med‐X Research Institute Shanghai Jiao Tong University Shanghai China

**Keywords:** mitochondria, mitochondrial DNA, Pentamidine, prostate cancer

## Abstract

**Objectives:**

We investigated the anti‐cancer activity of pentamidine, an anti‐protozoal cationic aromatic diamidine drug, in prostate cancer cells and aimed to provide valuable insights for improving the efficacy of prostate cancer treatment.

**Materials and methods:**

Prostate cancer cell lines and epithelial RWPE‐1 cells were used in the study. Cell viability, wound‐healing, transwell and apoptosis assays were examined to evaluate the influences of pentamidine in vitro. RNA‐seq and qPCR were performed to analyse changes in gene transcription levels upon pentamidine treatment. Mitochondrial changes were assessed by measuring mitochondrial DNA content, morphology, membrane potential, cellular glucose uptake, ATP production and ROS generation. Nude mouse xenograft models were used to test anti‐tumour effects of pentamidine in vivo.

**Results:**

Pentamidine exerted profound inhibitory effects on proliferation, colony formation, migration and invasion of prostate cancer cells. In addition, the drug suppressed growth of xenograft tumours without exhibiting any obvious toxicity in nude mice. Mechanistically, pentamidine caused mitochondrial DNA content reduction and induced mitochondrial morphological changes, mitochondrial membrane potential dissipation, ATP level reduction, ROS production elevation and apoptosis in prostate cancer cells.

**Conclusions:**

Pentamidine can efficiently suppress prostate cancer progression and may serve as a novel mitochondria‐targeted therapeutic agent for prostate cancer.

## 1 INTRODUCTION

According to 2018 global cancer statistics, prostate cancer is one of the most common cancers and a major cause of cancer‐related mortality in men worldwide.^1^ Radical prostatectomy, radiotherapy and castration (medical or surgical) therapies are available treatment options for prostate cancer, and next‐generation hormone therapeutics with abiraterone and enzalutamide can improve outcomes of metastatic castration‐resistant prostate cancer (CRPC).^2^, ^3^, ^4^, ^5^ However, drug resistance, low cure rates and poor prognosis remain as crucial issues waiting for solutions.^2^ Exploring new drugs, therapies and anti‐cancer mechanism is urgently needed for improving the efficacy of prostate cancer treatment.

Mitochondria play a critical role in cell survival and apoptosis, as they are key organelles for energy production.^6^ Mitochondrial structural changes and dysfunction have been associated with various disorders, especially neurodegenerative diseases and cancer.^6^, ^7^, ^8^ Deregulated mitochondrial metabolism has been proposed to have relevant effects on prostate carcinogenesis.^9^, ^10^, ^11^ Recent research indicates that high mitochondria content is associated with prostate cancer disease progression.^12^ Mitochondria are the only organelles possessing their own DNA besides the nucleus in animal cells. Human mitochondrial DNA (mtDNA) consists of 37 genes coding for 13 proteins, which are subunits of mitochondrial electron transport chain complexes, 22 transfer RNAs (tRNAs) and two subunits of ribosomal RNA (rRNA).^13^ Disruption of the mitochondrial replication or transcription machinery results in mitochondrial dysfunction with ensuing energetic insufficiency causing growth inhibition, ageing and even apoptosis.^14^ Cells that lack mitochondrial gene expression become more sensitive to apoptosis induction.^15^ The characteristics of mtDNA, lack of histone protection and limited repair capacity, make it an attractive target for tumour treatment. Therefore, an increasing number of anti‐cancer drugs targeting mitochondria and their DNA are under development.^16^, ^17^, ^18^

Pentamidine, a cationic aromatic diamidine drug, has been used clinically for the treatment of African trypanosomiasis, antimonial‐resistant leishmaniasis and babesiosis as well as the prophylaxis of pneumocystis carinii pneumonia in acquired immune deficiency syndrome (AIDS) patients for several decades.^19^, ^20^, ^21^, ^22^, ^23^ While how the medication works is not entirely clear, it was found to rapidly localize to mitochondria and trigger disruption of mitochondrial membrane potential (ΔΨm) in parasite cells.^24^, ^25^, ^26^ In addition, pentamidine can bind specifically and strongly in the DNA minor groove at AT sequences and induce destruction in mtDNA of parasite kinetoplasts, which causes cell death.^27^, ^28^, ^29^ In recent years, although using pentamidine as an anti‐tumour drug has been proposed,^30^, ^31^, ^32^, ^33^, ^34^ effects of pentamidine on prostate cancer are still poorly studied.

In this study, we identify pentamidine as a potent agent to inhibit prostate cancer. Pentamidine effectively represses proliferation, migration and invasion as well as induces apoptosis of prostate cancer cells. Moreover, pentamidine causes mtDNA reduction, mitochondrial morphological changes and dysfunction, which may serve as its anti‐tumour underlying mechanism.

## 2 MATERIALS AND METHODS

### 2.1 Cell culture and treatment

PC3, DU145, LAPC4, LNCaP and RWPE‐1 cell lines were purchased from ATCC. The cell lines have been recently authenticated by short tandem repeat (STR) profiling at the Shanghai Biowing Applied Biotechnology Company. PC3 and DU145 cells were cultured in Dulbecco's modified Eagle's medium (DMEM; Gibco) containing 10% foetal bovine serum (FBS), 100 U/mL penicillin and 100 μg/mL streptomycin. LAPC4 and LNCaP cells were cultured in RPMI‐1640 medium (Gibco) supplemented with 10% FBS, 100 U/mL penicillin and 100 μg/mL streptomycin. RWPE‐1 cells were cultured in keratinocyte serum‐free medium (K‐SFM, Gibco) supplemented with growth supplement provided with the K‐SFM kit, 100 U/mL penicillin and 100 μg/mL streptomycin. The culture medium was refreshed every 2 days. In experimental cultures, cells were treated with indicated concentrations of pentamidine (Selleck, S4007) or 1.5 mmol/L *N*‐acetyl‐l‐cysteine (NAC; Sigma, A9165). Sterile distilled water was served as the vehicle for pentamidine, and dimethyl sulfoxide (Sigma, D2650) was used as the solvent for NAC. NAC was administered with pentamidine at the same time. Other chemical reagents used in this study are listed in Table S1.

### 2.2 Cell viability and colony formation assays

Cells were maintained in 96‐well plates (2000 or 3000 cells/well) with 200 μL medium containing different doses of experimental drugs at 37°C for corresponding hours, following which 10 μL of Cell Counting Kit (CCK‐8; YEASEN and 40203ES80) was added to each well, and the plates were further incubated at 37°C in a humidified 5% CO_2_ atmosphere for 3‐4 hours. Finally, the absorbance at 450 nm was measured by a microplate reader (BioTek Synergy HT). For the colony formation assays, cells were seeded in 6‐well plates (1000 cells/well) and cultured with vehicle, 2.5 μmol/L pentamidine or 5 μmol/L pentamidine at 37°C for 48 hours. Then, the medium was replaced with fresh drug‐free medium and the cells were cultured continuously for 10 days (PC3 and DU145) or 15 days (LAPC4). Finally, the cells were fixed in 4% paraformaldehyde, stained with crystal violet and photographed.

### 2.3 Cell cycle analysis

Cells were cultured with vehicle or 2.5 μmol/L pentamidine in 6‐well plates (10^6^ cells/well) at 37°C for 24 hours and then harvested for propidium iodide (PI) staining, using the Cell Cycle Staining Kit (MultiSciences, CCS01) according to the manufacturer's instructions. Finally, the cells were analysed on a C6 flow cytometer (BD Biosciences).

### 2.4 Wound‐healing assays and transwell experiments

Cells were pre‐treated with 2.5 μmol/L pentamidine or vehicle for 48 hours and seeded into 12‐well culture plates (5 × 10^5^ cells/well). When the cells reached confluence, the culture medium was replaced with DMEM medium without serum to minimize cell proliferation. Then, a pipette tip was used to make a straight scratch. The scratch was examined and photographed under a light microscope at 0, 24, 48 and 72 hours. Cell‐free area was quantified by the ImageJ software. For the transwell migration experiments, 2 × 10^4^ cells were seeded into the upper chamber of 24‐well transwell plates (6.5 mm insert, 8.0 μm pores) with 200 μL serum‐free medium. 600 μL 10% FBS‐supplemented medium was added to the lower chamber. After 15 hours, cells were removed from the upper surface of the chamber using a cotton swab. The migrated cells on the chamber bottom were then fixed with 4% paraformaldehyde and stained with crystal violet. For the transwell invasion experiments, 50 µl of diluted Matrigel (BD, 356234) was pipetted into the upper chamber of 24‐well transwell plates. The Matrigel was incubated at 37°C, 5% CO2 for 45 minutes prior to addition of cells to the chamber. 4 × 10^4^ cells were seeded into the upper chamber with 200 μL serum‐free medium. 600 μL 10% FBS‐supplemented medium was added to the lower chamber. After 20 hours, the Matrigel and non‐invading cells were gently removed from the upper surface of the chamber using a cotton‐tipped swab. The cells on the chamber bottom were then fixed and stained for visualization. Three fields per chamber were photographed under a light microscope for quantification.

### 2.5 mRNA sequencing analysis

Total RNA was extracted from cells treated with 2.5 μmol/L pentamidine or vehicle for 48 hours using the TRIzol reagent (Invitrogen, 15596018), and mRNA was enriched by Oligo (dT) beads. Then, the enriched mRNA was fragmented into short fragments using the fragmentation buffer and reverse transcribed into cDNA with random primers. Second‐strand cDNA fragments were synthesized by DNA polymerase I, RNase H, dNTP and buffer. Then, these cDNA fragments were purified with QiaQuick PCR extraction kit, end repaired, poly(A) added and ligated to Illumina sequencing adapters. The ligation products were then size selected by agarose gel electrophoresis, PCR amplified and sequenced using Illumina Novaseq 6000 PE150. Paired‐end clean reads were aligned to the reference genome using HISAT2 (v2.1.0). Differential expression analysis of two groups was performed using the DESeq R package (1.18.1). The resulting *P*‐values were adjusted using Benjamini and Hochberg's approach for controlling the false discovery rate. Genes with |log_2_(FoldChange)| > 1 and adjusted *P* value <.05 found by DESeq were assigned as differentially expressed. This experiment was conducted by Haplox Biotechnology Co. (ShenZhen, China). Gene set enrichment analysis (GSEA) was performed using the java GSEA software. The RNA sequencing (RNA‐seq) data set was submitted to the GEO database with the accession number GSE132693.

### 2.6 Quantitative PCR assays

Total RNA was isolated from cells pre‐treated with 2.5 μmol/L pentamidine or vehicle for 48 hours using the TRIzol reagent (Invitrogen, 15596018) and then reverse transcribed to cDNA using the PrimeScript RT Reagent Kit (Takara, RR037A) according to the manufacturer's instructions. Quantitative polymerase chain reaction (qPCR) was performed using the TB Green Premix Ex Taq (Takara, RR420A) and the Step one Plus Real‐Time PCR System (Applied Biosystems, Waltham). The relative expression of mRNA was normalized to the expression of β‐actin and analysed using the 2^−ΔΔCт^ method. All experiments were repeated three times. qPCR primer sequences used in this study are shown in Table S2.

### 2.7 mtDNA content analysis

The mtDNA content in cells pre‐treated with 2.5 μmol/L pentamidine or vehicle for 48 hours was analysed by qPCR as previously described.^35^, ^36^ Briefly, total DNA was extracted using the QIAamp DNA Micro kit (Qiagen, 56304) and qPCR reactions were performed on the Step one Plus Real‐Time PCR System (Applied Biosystems, Waltham) according to manufacturer's protocols. The sequences of the primers were as follows: mtDNA (5′‐CCC CAC AAA CCC CAT TAC TAA ACC CA‐3′; 5′‐TTT CAT CAT GCG GAG ATG TTG GAT GG‐3′) and β‐globin (5′‐CGA GTA AGA GAC CAT TGT GGC AG‐3′; 5′‐GCT GTT CTG TCA ATA AAT TTC CTTC‐3′). The mtDNA content was normalized to the expression of β‐globin and analysed using the 2^−ΔΔCт^ method.

### 2.8 Mitochondrial morphology analysis

Cells were cultured with vehicle or 2.5 μmol/L pentamidine in 6‐well plates (10^6^ cells/well) at 37°C for 48 hours and then washed, harvested and fixed at 4°C for 24 hours with Fixing Solution (Servicebio, G1102). The cells were then post‐fixed in 1% osmium tetroxide, dehydrated in a graded series of ethanol, infiltrated and embedded in EMBed. Ultrathin sections were evaluated using a HT7700 transmission electron microscope (HITACHI). To observe the mitochondrial network changes, cells pre‐treated with 2.5 μmol/L pentamidine or vehicle for 48 hours were stained with 100 nmol/L MitoTracker Deep Red FM (Invitrogen, M22426) at 37°C for 30 minutes and then washed, fixed, stained with 4′,6‐diamidino‐2‐phenylindole (DAPI), captured by a LSM710 confocal microscope (Carl Zeiss, Jena) and analysed using ImageJ software. Mitochondria were subjected to “analyse particles” to obtain the mitochondrial elongation (ratio of the lengths of major and minor axes) and the mitochondrial interconnectivity (ratio of the area and the perimeter), two mediators of mitochondrial fission and fusion as described before.^37^ More than 50 cells were measured in each group.

### 2.9 Mitochondrial membrane potential and ATP synthesis detection

Live cells were labelled with tetraethylbenzimidazolylcarbocyanine iodide (JC‐1, MultiSciences, MJ101), and the ΔΨm was measured by flow cytometry (BD Biosciences). JC‐1 is a cationic dye that accumulates in energized mitochondria driven by ΔΨm. When ΔΨm is relatively normal, JC‐1 tends to gather in the mitochondria and form red‐fluorescent aggregate, whereas it is prone to release from mitochondria and exist as green‐emitting monomer in the cytosol when ΔΨm is decreased.^38^, ^39^ Consequently, disruption of ΔΨm is indicated by a loss of red fluorescence as well as an increase in green fluorescence. Cells pre‐treated with 2.5 μmol/L pentamidine or vehicle for 48 hours were incubated with 2 μmol/L JC‐1 for 30 minutes at 37°C. Then, the treated cells were washed, collected and resuspended in 200 μL PBS buffer for flow cytometric analysis. ΔΨm was evaluated by the JC‐1 aggregate/monomer fluorescence ratio. For the ATP synthesis detection, cells were seeded into 6‐well plates (10^6^ cells/well), treated with vehicle or 2.5 μmol/L pentamidine at 37°C for 48 hours and finally collected for ATP determination using an ATP assay kit (Nanjing Jiancheng, A095). In addition, glucose concentrations of the medium were detected by a glucose assay kit (Rsbio, 361510).

### 2.10 Assessments of apoptosis and intracellular ROS production

Cells were cultured with vehicle or 2.5 μmol/L pentamidine in 6‐well plates (10^6^ cells/well) at 37°C for 48 hours and then harvested for Annexin V‐APC (Biolegend, 640920) and PI (Biolegend, 421301) staining. Finally, the cells were analysed on a flow cytometer (BD Biosciences). Reactive oxygen species (ROS) production was determined using 2′,7′‐dichlorofluorescein diacetate (Sigma, 35845). Cells pre‐treated with vehicle, 2.5 μmol/L pentamidine or 1.5 mmol/L NAC for 48 hours were incubated with 2 μmol/L 2′,7′‐dichlorofluorescein diacetate for 30 minutes at 37°C. Then, the treated cells were washed, collected and resuspended in 200 μL PBS buffer for flow cytometric analysis. ROS levels were evaluated by the fluorescence intensity of 2′,7′‐dichlorofluorescein diacetate.

### 2.11 Animal studies

Animal experiments were carried out following protocols approved by Ren Ji Hospital's committee on animal care. Five‐week‐old male athymic nude mice (Shanghai SLAC Laboratory Animal) were subcutaneously injected with 3 × 10^6^ PC3 or DU145 cells and treated with pentamidine (20 mg/kg) or vehicle by intraperitoneal injection once every three days from the eighth day after implantation. The solvent for pentamidine was sterile water for in vivo studies. Tumour volume and body weight of nude mice were measured periodically in the next two weeks after pentamidine administration. Tumour volume was estimated from the formula: *V* = *L* × *W* ^2^/2 (*V*, volume, mm^3^; *L*, major axis length, mm; *W*, minor axis length, mm). Lengths of major and minor axes were measured with a digital caliper. After 2‐week drug administration, the tumours were harvested, weighed, imaged, fixed with 4% paraformaldehyde and made into paraffin sections.

### 2.12 Immunohistochemistry assays

Immunohistochemistry was performed using the xenograft tumour paraffin sections. The Instant Immunohistochemistry Kit I (Sangon, C506333), anti‐Ki67 antibody (Abcam, ab15580), MTCO2 antibody (Proteintech, 55070‐1‐AP), cleaved caspase‐3 antibody (Cell Signaling Technology, 9661), HRP‐conjugated goat anti‐rabbit IgG (Sangon, D110073) and DAB Peroxidase Substrate Kit (Vector, SK‐4100) were used for immunohistochemistry. Stained sections were examined and photographed under a light microscope.

### 2.13 Western blot assays

Cells were cultured with vehicle or 2.5 μmol/L pentamidine in 6‐well plates (10^6^ cells/well) at 37°C for 24 hours and then lysed using RIPA Lysis and Extraction Buffer (Thermo Scientific, 89901) containing the protease inhibitor cocktail (Thermo Scientific, 87786). Proteins were then separated by SDS‐polyacrylamide gel electrophoresis and transferred to nitrocellulose membranes. After blocked with 5% fat‐free milk for 1 hour at room temperature, the membranes were incubated with primary antibodies at 4°C overnight: P21 antibody (Proteintech, 10355‐1‐AP), P53 antibody (Proteintech, 10442‐1‐AP) and beta‐actin antibody (Proteintech, 7D2C10). The membranes were then incubated with horseradish peroxidase‐conjugated secondary antibodies for 1 hour at room temperature: Goat anti‐rabbit IgG (Proteintech, SA00001‐2) and goat anti‐mouse IgG (Proteintech, SA00001‐1).

### 2.14 Statistical analysis

GraphPad Prism 7 software was used for graphical and statistical analyses. Experimental results were expressed as mean ± SD. Student's *t* test was used for statistical analysis. *P* values <.05 (*), <.01 (**) or <.001 (***) were considered to have statistical significance.

## 3 RESULTS

### 3.1 Pentamidine inhibits prostate cancer cell proliferation

**Figure 1:**
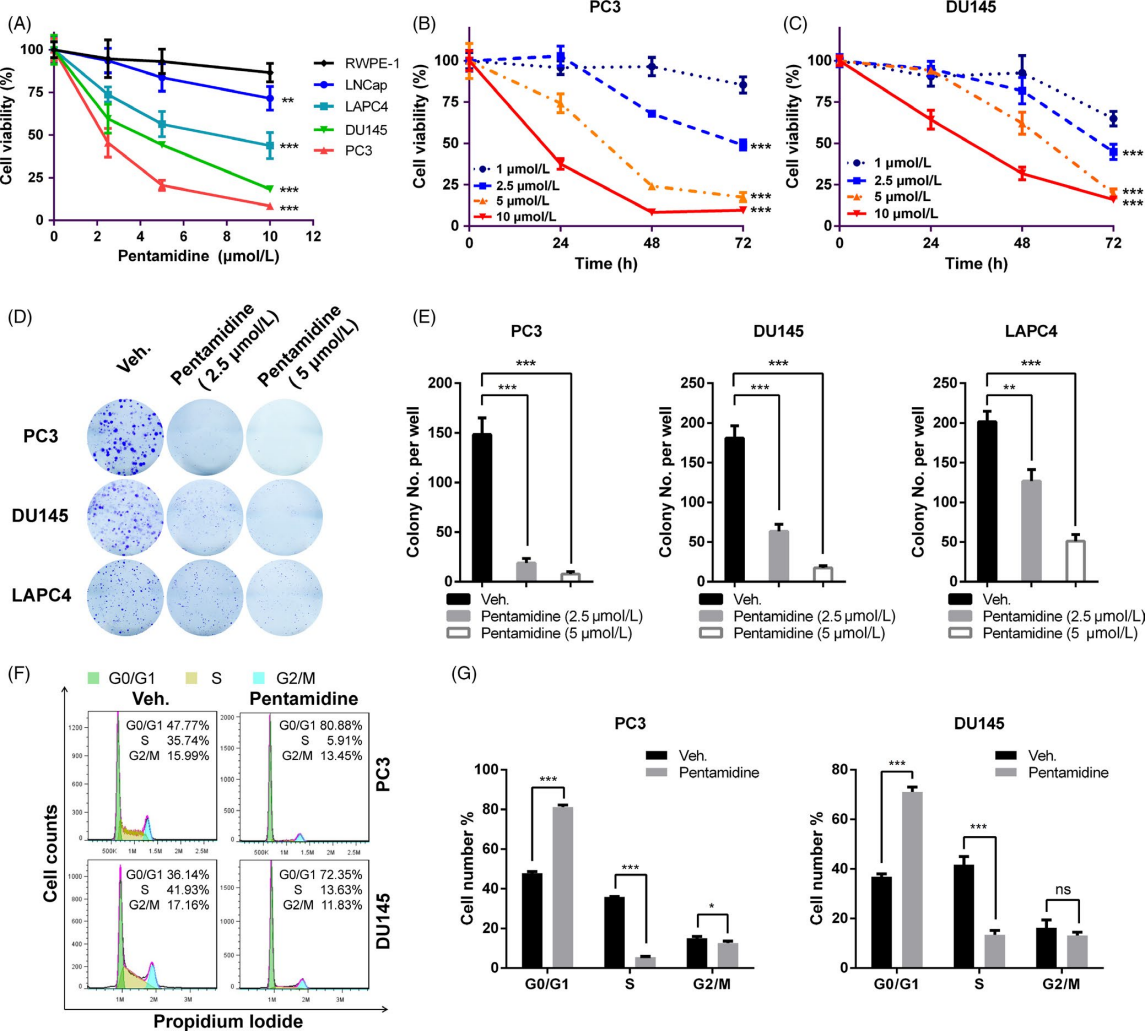
Pentamidine inhibits proliferation in prostate cancer cells. A, Cells were treated with pentamidine (0, 2.5, 5 and 10 μmol/L) for 48 h, and then, cell viability was determined by CCK‐8 assays. Statistical analysis was performed between each prostate cancer cell line and RWPE‐1. B, C, PC3 and DU145 cells were treated with pentamidine (1, 2.5, 5 and 10 μmol/L) for 0, 24, 48 or 72 h, and then, cell viability was determined by CCK‐8 assays. Statistical analysis was performed between cells treated with 1 μmol/L pentamidine and each of the other drug concentration groups. D, E, Long‐term colony formation assays of PC3, DU145 and LAPC4 cells induced by pentamidine (0, 2.5 and 5 μmol/L). (F‐G) Cell cycle distribution of PC3 and DU145 cells incubated with 2.5 μmol/L pentamidine or vehicle for 24 h. Unpaired *t* test was used for the statistical analysis. *P < .05; **P < .01; ***P < .001; ns, no significance. Data are presented as mean ± SD of at least three independent experiments

To determine whether pentamidine has any effect on prostate cancer cell growth, we first examined the cell viability of prostate cancer cells upon pentamidine treatment by CCK‐8 assays and found that the androgen‐independent prostate cancer cells (PC3 and DU145) were more sensitive to pentamidine than androgen‐dependent LAPC4 and LNCap cells, whereas the drug displayed slight influence on cell vitality of RWPE‐1, which is a normal human prostate epithelial cell line (Figure 1A). Specifically, pentamidine exerted a significant inhibition on cell proliferation of PC3 and DU145 cells in a dose‐dependent as well as time‐dependent manner (Figure 1B‐C). In addition, pentamidine was found to markedly suppress colony formation ability of prostate cancer cells in a dose‐dependent manner (Figure 1D‐E).

To explore whether pentamidine caused the arrest of cell cycle, a flow cytometric analysis of PC3 and DU145 stained with PI was performed. We found that pentamidine caused a significant increase in population of the G0/G1 cells along with a loss of cells in S phase, which intimated a block in G1/S progression (Figure 1F‐G). Furthermore, Western blot confirmed the increase in expression level of p53 and p21 in PC3 and DU145 cells under pentamidine treatment (Figure S1A). Taken together, these results suggest that pentamidine has potent anti‐tumour effects on prostate cancer cells.

### 3.2 Pentamidine suppresses the migration and invasion of prostate cancer cells

**Figure 2:**
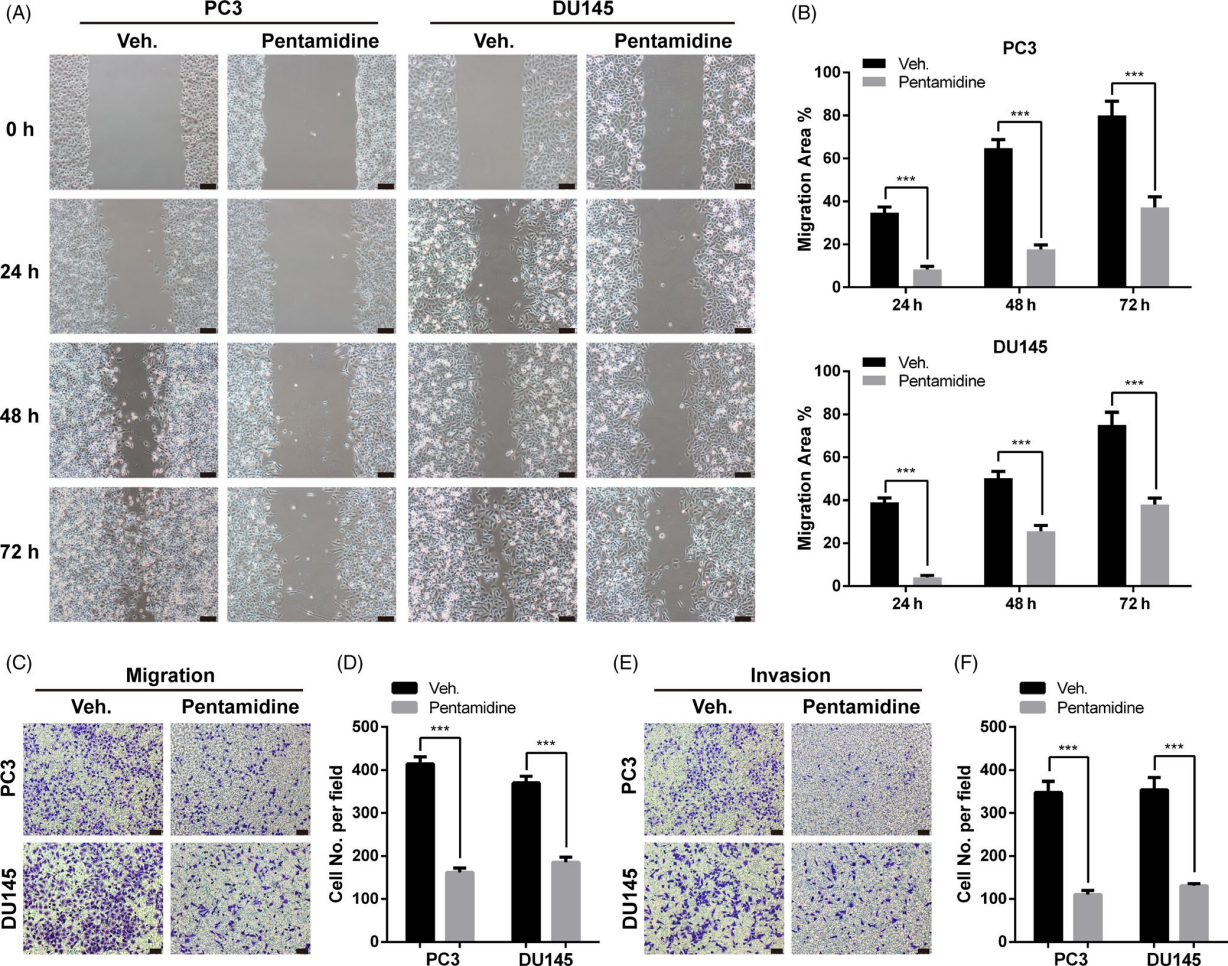
Pentamidine suppresses the migration and invasion of prostate cancer cells. A, B, Wound‐healing assays of PC3 and DU145 cells pre‐treated with 2.5 μmol/L pentamidine or vehicle. Scale bar, 100 μm. C, D, Transwell migration experiments of PC3 and DU145 cells pre‐treated with 2.5 μmol/L pentamidine or vehicle. Scale bar, 100 μm. (E‐F) Transwell invasion experiments of PC3 and DU145 cells pre‐treated with 2.5 μmol/L pentamidine or vehicle. Scale bar, 100 μm. Unpaired *t* test was used for the statistical analysis. ***P < .001. Data are presented as mean ± SD of at least three independent experiments

We next determined whether pentamidine exhibited an inhibitory role on migration in prostate cancer cells. As characterized by wound‐healing assays, pentamidine treatment led to a marked reduction in migration ability of PC3 and DU145 cells (Figure 2A, B). Similarly, transwell migration experiments indicated the inhibitory effects of pentamidine on cell mobility (Figure 2C, D). To evaluate the effects of pentamidine on invasion of prostate cancer cells, we performed transwell invasion experiments and found that the invasion ability of tumour cells was much lower in pentamidine treatment groups (Figure 2E, F). These results suggest that pentamidine inhibits prostate cancer cell migration and invasion in vitro.

### 3.3 Pentamidine causes mtDNA reduction in prostate cancer cells

**Figure 3:**
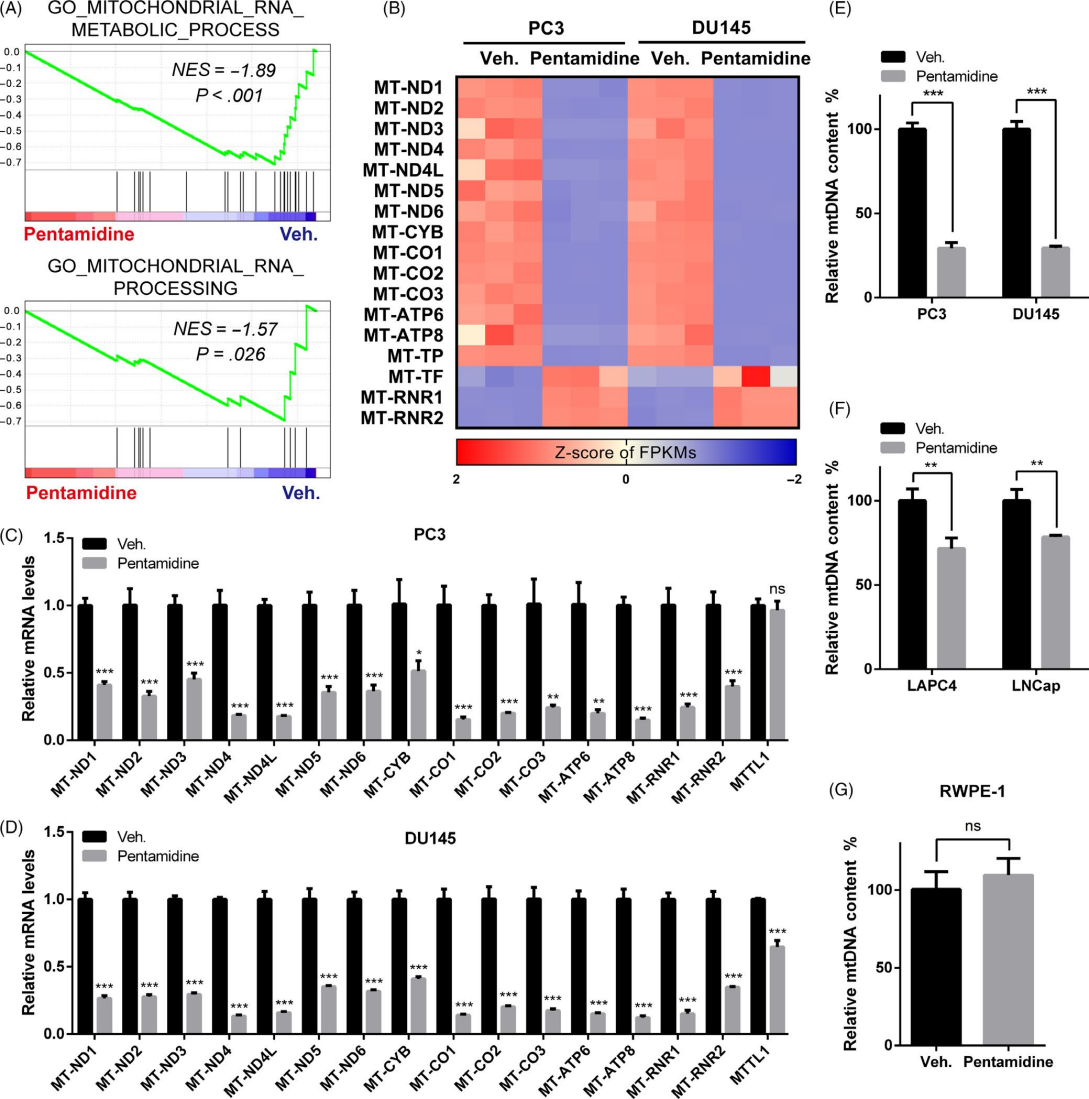
Pentamidine causes mtDNA reduction in prostate cancer cells. A, GSEA analysis of RNA‐seq data indicated downregulation of genes involved in processes related to mitochondrial RNA and its metabolism in pentamidine‐treated prostate cancer cells. NES, normalized enrichment score. B, Heatmap of the mRNA expression of mitochondria‐encoded genes in pentamidine‐ and vehicle‐treated prostate cancer cells, as analysed by RNA‐seq. FPKMs, fragments per kilobase of transcript per million mapped reads. C, D, qPCR analysis of transcription levels of mitochondria‐encoded genes in PC3 and DU145 cells. E‐G Effects of pentamidine on mtDNA content in PC3, DU145, (E) LAPC4, LNCap, (F) and RWPE‐1 (G) cells. mtDNA, mitochondrial DNA. Unpaired *t* test was used for the statistical analysis. *P < .05; **P < .01; ***P < .001; ns, no significance. Data are presented as mean ± SD of at least three independent experiments

To uncover the mechanism underlying the inhibitory effects of pentamidine on prostate cancer cells, we performed RNA‐seq to compare the transcriptional difference between pentamidine‐ and vehicle‐treated PC3 as well as DU145 cells. GSEA of RNA‐seq data indicated suppression of processes related to mitochondrial RNA and its metabolism in cells upon pentamidine treatment (Figure 3A). Interestingly, the transcription levels of most of the mitochondria‐encoded genes were significantly reduced (Figure 3B). Furthermore, qPCR assays verified the reduced expression of related genes in PC3 and DU145 cells (Figure 3C‐D). The transcription levels of TFAM and NRF1, encoding transcription factors in charge of mitochondrial transcription, had no significant change in PC3 and DU145 cells upon pentamidine treatment (Figure S1B).

Pentamidine was previously reported to bind to and break mtDNA in parasites.^27^, ^28^, ^29^ Hence, we investigated whether pentamidine decreased mtDNA content in prostate cancer cells. We observed that pentamidine treatment indeed caused mtDNA reduction. In detail, pentamidine exhibited a significant decrease in PC3 and DU145 mtDNA content (Figure 3E), whereas the reduction in mtDNA was moderate in LNCap and LAPC4 cells (Figure 3F), which further verified that pentamidine was more suitable for targeting the androgen‐independent prostate cancer cells. However, the mtDNA level in RWPE‐1 cells was approximately not affected (Figure 3G). Collectively, these results indicate that pentamidine selectively impairs the mtDNA of prostate cancer cells.

### 3.4 Pentamidine induces mitochondrial morphological changes and dysfunction

**Figure 4:**
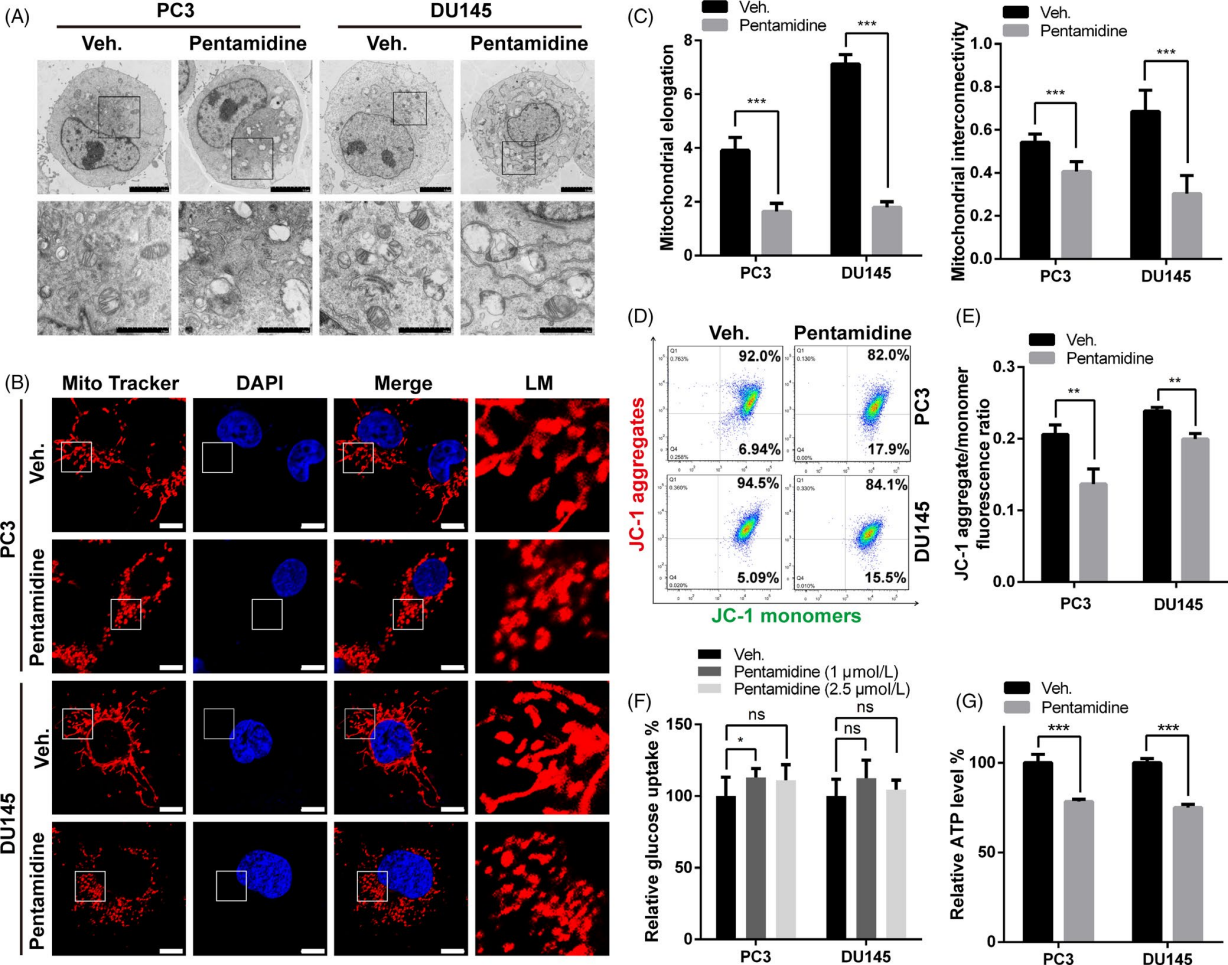
Pentamidine induces mitochondrial morphological changes and dysfunction. A, Transmission electron micrographs of PC3 and DU145 cells upon 2.5 μmol/L pentamidine or vehicle treatment. Scale bar, 5 μm (top), 2 μm (bottom). B, Effects of pentamidine on mitochondrial network morphology in PC3 and DU145 cells stained with MitoTracker Red. Scale bar, 10 μm. LM, local magnification. C, Quantitative image analysis of mitochondrial elongation and interconnectivity by ImageJ. D‐E, Flow cytometric analysis of ΔΨm in PC3 and DU145 cells treated with 2.5 μmol/L pentamidine or vehicle. ΔΨm was evaluated by the JC‐1 aggregate/monomer fluorescence ratio. F, Effects of pentamidine on glucose uptake in PC3 and DU145 cells. G, Influences of pentamidine in ATP production of PC3 and DU145 cells. Unpaired t test was used for the statistical analysis. *P < .05; **P < .01; ***P < .001; ns, no significance. Data are presented as mean ± SD of at least three independent experiments

Transmission electron micrographs revealed that mitochondria of pentamidine‐treated cells were swollen and distensible. In vehicle‐treated cells, we found many mitochondria with flattened cristae, whereas the cristae system practically disappeared in swollen mitochondria of PC3 and DU145 cells upon pentamidine treatment (Figure 4A). We further explored the influence on mitochondrial network of pentamidine in PC3 and DU145 cells. In control groups, the mitochondrial network was interconnected and extensive throughout the cells (Figure 4B). However, in pentamidine‐treated cells, the mitochondrial network was significantly disturbed and scattered (Figure 4B), accompanied by average mitochondrial elongation, and interconnectivity values were significantly decreased (Figure 4C), which suggested the mitochondrial network fragmentation.^37^

We labelled live cells with JC‐1 and measured the ΔΨm.^38^, ^39^ The data demonstrated that pentamidine treatment dissipated ΔΨm in PC3 and DU145 cells (Figure 4D, E). Next, we determined the energy production ability of mitochondria and found that glucose uptake increased slightly, but not significantly; whereas there was a decrease in the ATP level of both cell lines upon pentamidine treatment (Figure 4F, G). These data indicate that pentamidine induces mitochondrial morphological changes and dysfunction in prostate cancer cells.

### 3.5 Pentamidine leads to apoptosis in prostate cancer cells

**Figure 5:**
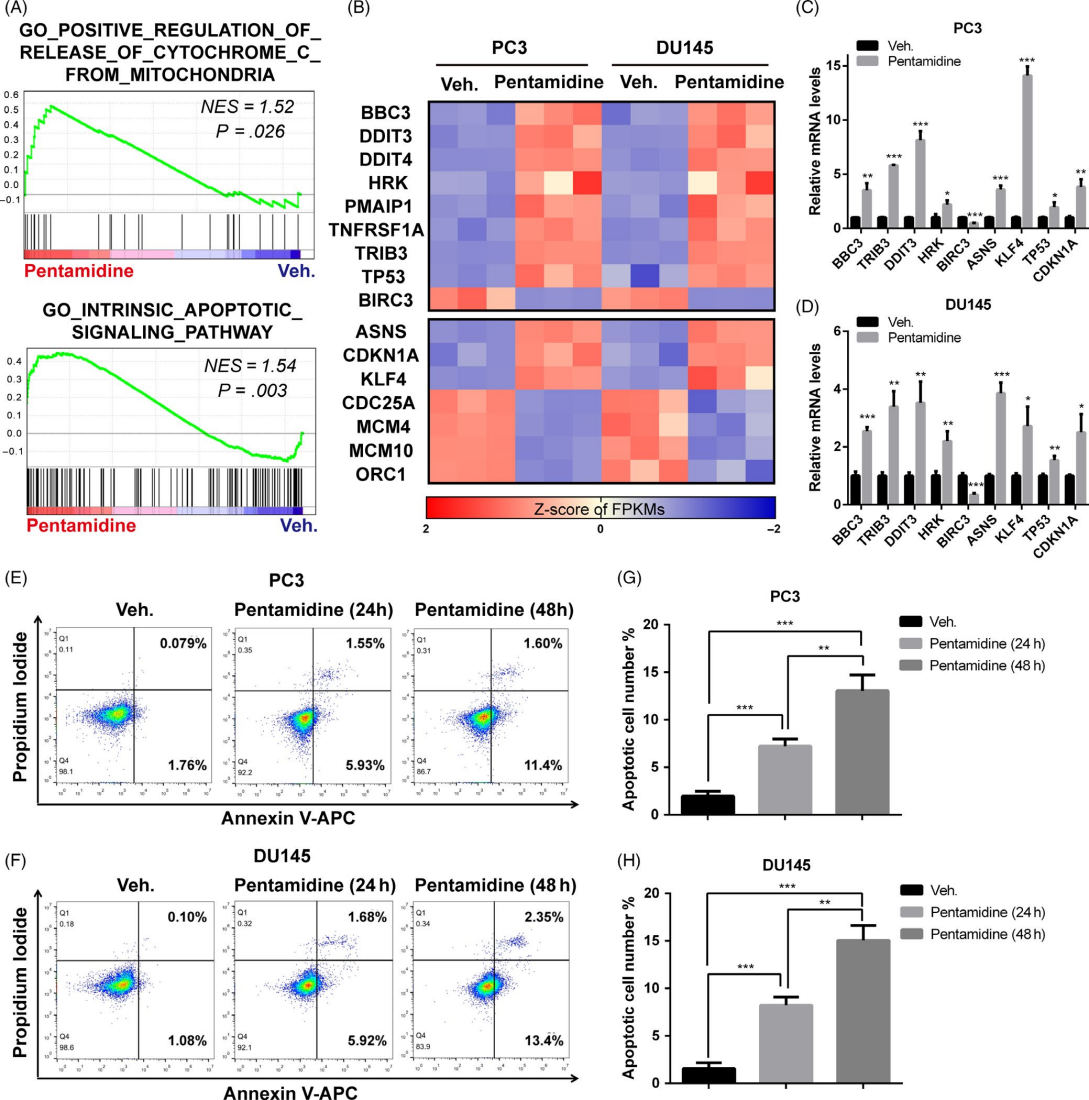
Pentamidine leads to apoptosis in prostate cancer cells. A, GSEA analysis of RNA‐seq data indicated a positive regulation of release of cytochrome c from mitochondria and intrinsic apoptotic signalling pathway in prostate cancer cells upon pentamidine treatment. NES, normalized enrichment score. B, Heatmaps of the mRNA expression of genes related to apoptosis and cell cycle in pentamidine‐ and vehicle‐treated PC3 and DU145 cells, as analysed by RNA‐seq. FPKMs, fragments per kilobase of transcript per million mapped reads. C, D, qPCR analysis of transcription levels of genes related to apoptosis and cell cycle in PC3 and DU145 cells. E‐H, Flow cytometric assays of the apoptotic percentage (including viable and non‐viable apoptotic cells) in pentamidine‐ and vehicle‐treated PC3 and DU145 cells. Unpaired *t* test was used for the statistical analysis. *P < .05; **P < .01; ***P < .001. Data are presented as mean ± SD of at least three independent experiments

When we examined the morphology of prostate cancer cells under light microscopes, pentamidine was found to induce morphological changes associated with cell death (Figure S1C). Mitochondria have a major role in apoptosis induction. Disruption of ΔΨm is accompanied by the translocation and release of apoptogenic factors, including cytochrome c, which then activate mitochondrial‐mediated apoptosis pathways. GSEA analysis of RNA‐seq data indicated a positive regulation of release of cytochrome c from mitochondria and intrinsic apoptotic signalling pathway in pentamidine‐treated prostate cancer cells (Figure 5A). Genes coding for pro‐apoptotic proteins, such as BBC3, TRIB3, DDIT3 and HRK, were shown to be significantly upregulated at mRNA levels in prostate cancer cells upon pentamidine treatment, whereas the transcription level of BIRC3, which encoded an inhibitor of apoptosis, was downregulated in pentamidine‐treated PC3 and DU145 cells (Figure 5B). These results were verified by qPCR (Figure 5C‐D). Data of RNA‐seq and qPCR also indicated an arrest of cell cycle phase transition (Figure 5B, D; Figure S1D), which was consistent with our previous experimental results (Figure 1F, G). Next, we performed cell apoptosis assays using an Annexin V‐APC/PI double staining kit and flow cytometry analysis, which confirmed that pentamidine markedly induced cell apoptosis in PC3 and DU145 cells (Figure 5E‐H).

### 3.6 Pentamidine mediates prostate cancer cell apoptosis by inducing ROS production

**Figure 6:**
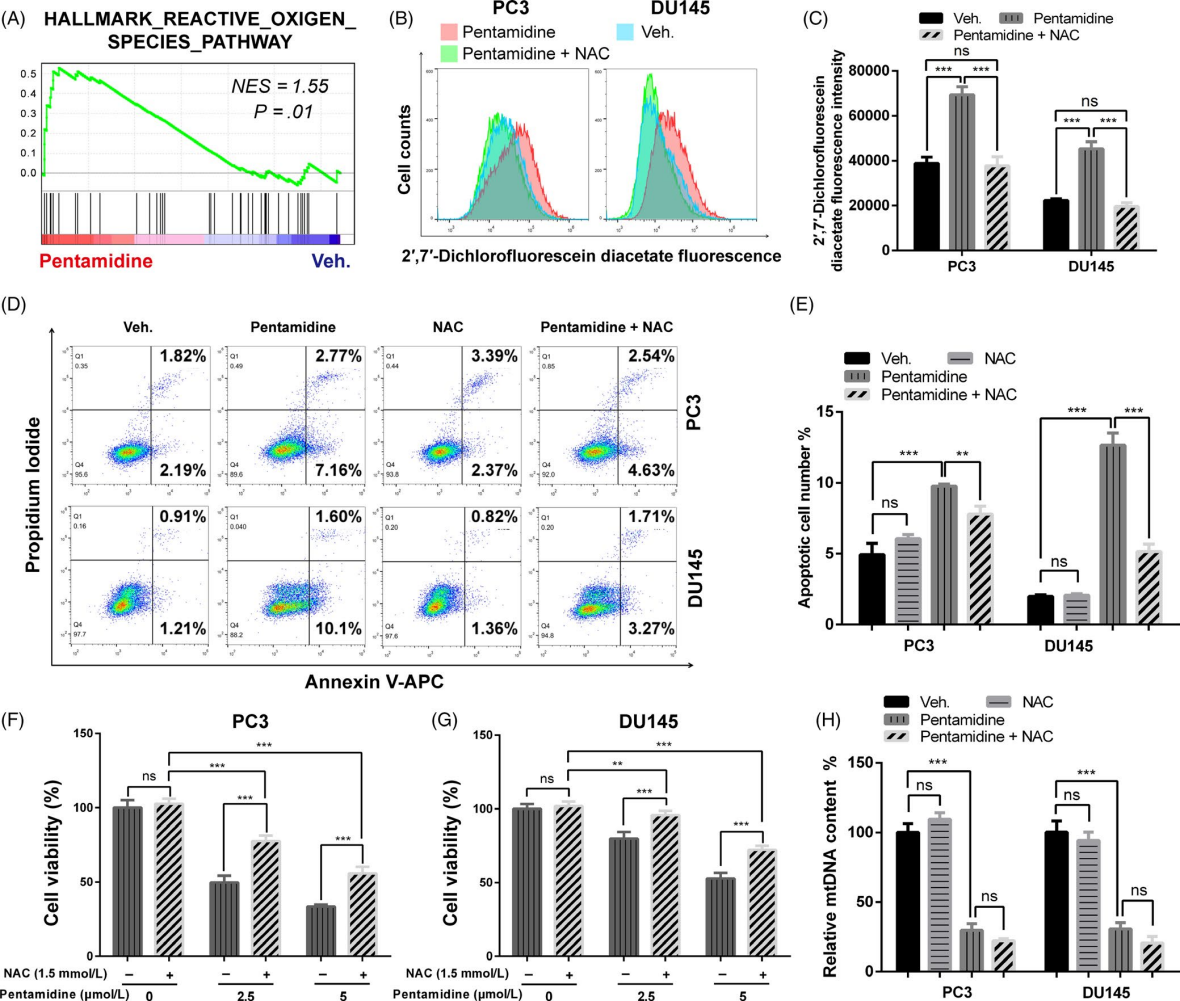
Pentamidine mediates prostate cancer cell apoptosis by inducing ROS production. A, GSEA analysis of RNA‐seq data indicated the activation of ROS pathway in pentamidine‐treated prostate cancer cells. NES, normalized enrichment score. B, C, Effects of pentamidine and NAC on ROS levels. ROS production was evaluated by 2′,7′‐dichlorofluorescein diacetate fluorescence intensity. NAC, *N*‐acetyl‐l‐cysteine. D, E, Flow cytometric assays of the apoptotic percentage (including viable and non‐viable apoptotic cells) in PC3 and DU145 cells upon vehicle, pentamidine or NAC treatment. F, G, Cell viability of PC3 and DU145 cells treated with pentamidine (0, 2.5 and 5 μmol/L) or 1.5 mmol/L NAC for 48 h. H, Effects of NAC on mtDNA content in PC3 and DU145 cells upon 2.5 μmol/L pentamidine or vehicle treatment. mtDNA, mitochondrial DNA. Unpaired t test was used for the statistical analysis. **P < .01; ***P < .001; ns, no significance. Data are presented as mean ± SD of at least three independent experiments

ROS generation plays a key role in regulating DNA damage and apoptosis.^40^ GSEA analysis of RNA‐seq data indicated an activation of ROS pathway in pentamidine‐treated prostate cancer cells (Figure 6A). We then examined the intracellular ROS levels by assessing the fluorescence intensity of 2′,7′‐dichlorofluorescein diacetate dye. Compared with the control cells, prostate cancer cells exposed to pentamidine showed a higher 2′,7′‐dichlorofluorescein diacetate fluorescence intensity, indicating that pentamidine enhanced ROS production in these cells (Figure 6B, C).

NAC, an antioxidant, was able to reduce ROS in PC3 and DU145 cells (Figure 6B, C). We found that NAC relieved the cell apoptosis and proliferation inhibition induced by pentamidine, whereas itself had no significant effect on apoptosis, proliferation and mtDNA content in prostate cancer cells (Figure 6D‐H). The results suggest that pentamidine induces prostate cancer cell apoptosis by upregulating ROS production. In addition, notably, reduction in mtDNA induced by pentamidine was not mitigated by NAC (Figure 6H).

### 3.7 Pentamidine inhibits tumour growth in vivo

**Figure 7:**
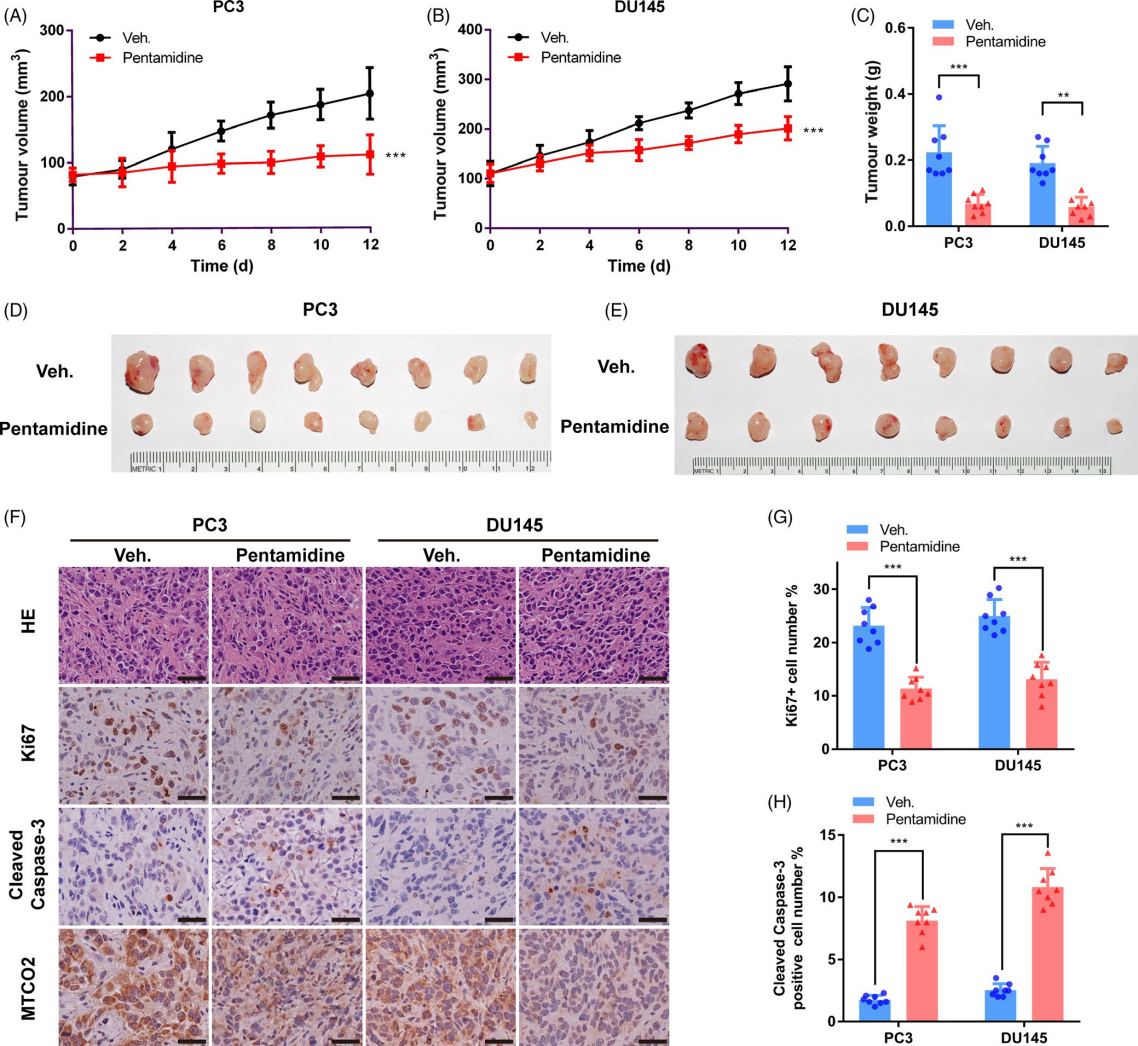
Pentamidine inhibits tumour growth in vivo. A, B Relative tumour volume in nude mice. Tumour volume was measured once every two days from the day of drug administration. C, Weight of xenograft tumours when they were harvested. D, E, Photographs of xenograft tumours when they were harvested. F, H&E staining and immunohistochemistry images with antibodies against Ki67, MTCO2 or cleaved caspase‐3 in PC3 and DU145 xenograft tumours. Scale bar, 50 μm. G, Proportion of Ki67 positive cells was reduced in xenograft tumours upon pentamidine treatment. H, Proportion of cleaved caspase‐3 positive cells was increased in xenograft tumours upon pentamidine treatment. Unpaired *t* test was used for the statistical analysis. **P < .01; ***P < .001. Data are presented as mean ± SD

We next tested whether pentamidine played an anti‐tumour effect in vivo using nude mouse xenograft models. Pentamidine was found to significantly suppress the growth of xenograft tumours (Figure 7A‐E). The reduction in tumour volume and weight was not a consequence of overall toxicity, as we did not observe body weight loss in mice treated with pentamidine (Figure S2A‐B). Immunohistochemistry assays showed that the levels of Ki67, a marker of proliferating cells, and MTCO2, a subunit of the cytochrome C oxidase encoded by the mitochondria, were reduced, and the proportion of cleaved caspase‐3 positive cells was significantly increased in xenograft tumours upon pentamidine treatment (Figure 7F‐H). Taken together, these results demonstrate that pentamidine inhibits prostate cancer growth as well as induces mitochondrion‐related changes and apoptosis in vivo.

## 4 DISCUSSION

In the current study, we investigated the anti‐cancer activity of an anti‐protozoal aromatic diamidine derivative, pentamidine, in prostate cancer cells. We find that pentamidine exerts a profound inhibitory effect on proliferation, colony formation, migration and invasion in prostate cancer cells. Systemic administration of pentamidine markedly suppresses the tumour growth capacity of prostate cancer in vivo. In addition, we further demonstrate that pentamidine causes mtDNA reduction and induces mitochondrial morphological changes, mitochondrial dysfunction, ROS generation and apoptosis in prostate cancer cells.

In the practice of developing efficacious anti‐cancer drugs, repositioning of current clinical drugs may serve as an attractive approach owing to their favourable in vivo safety and relatively clear pharmacokinetics and pharmacodynamics.^41^, ^42^ Dr Jung and colleagues found that pentamidine reduced expression of hypoxia‐inducible factor‐1α (HIF‐1α) in DU145 cells.^34^ In the present study, we examined the activity of pentamidine in several prostate cancer cells, including PC3, DU145, LAPC4 and LNCap, as well as normal prostate epithelial cells, RWPE‐1. Interestingly, PC3 and DU145 cells, which are androgen‐independent and do not express prostate‐specific antigen (PSA), are more sensitive to pentamidine than LAPC4 and LNCap cells. In addition, accompanied by its inhibitory effects on proliferation, migration and invasion of PC3 and DU145 cells and low toxicity to RWPE‐1 cells in vitro, pentamidine treatment achieves great anti‐tumour effects without obvious toxicity in nude mouse xenograft models. These results suggest a promising repositioning of pentamidine for the treatment of prostate cancer. However, the potential clinical application of pentamidine in broader tumour types needs to be further evaluated.

Mitochondria play a vital role in proliferation and apoptosis of a cell. mtDNA is necessary for respiratory function and tumorigenic potential of cancer cells.^43^, ^44^ Therefore, many research groups are trying to search anti‐cancer drugs targeting mitochondria and mtDNA.^16^, ^45^, ^46^ However, small‐molecule drug candidate with good in vivo tolerance and favourable pharmacodynamics are still lacking.^46^ Data presented here demonstrate that pentamidine may serve as a novel mitochondria‐targeted therapeutic agent for prostate cancer. RNA‐seq indicates that transcription levels of the vast majority of mitochondria‐encoded genes are significantly reduced upon pentamidine treatment, but MT‐RNR1 and MT‐RNR2 are increased. This discrepancy is presumably due to a small number of errors during high‐throughput sequencing, because the qPCR assays further verify the reduction in the transcription of all mitochondria‐encoded genes including MT‐RNR1 and MT‐RNR2. In addition, we have demonstrated that pentamidine directly causes mtDNA reduction, mitochondrial morphological and functional alterations, including mitochondrial swelling and enlargement, cristae system disappearance, ΔΨm dissipation and cytochrome C release from the mitochondria into the cytosol, which induce mitochondrial‐mediated apoptotic cell death.

In view of the importance of mitochondria and mtDNA in cell growth and survival, our current study provides a close link between the anti‐tumour effects of pentamidine and reduction in mtDNA based on following reasons. Firstly, previous studies have shown that pentamidine binds specifically and strongly to the DNA minor groove at AT sequences.^27^, ^28^, ^29^ The mtDNA is organized as circular double‐helical structure and contains extensive and closely spaced AT sequences, which provides potential cellular targets for pentamidine.^13^, ^47^ Secondly, the characteristics of pentamidine, cationic charge and lipophilicity allow it to harness the negative membrane potential and hydrophobic membranes of mitochondria and localize to them.^27^, ^46^ Thirdly, the increased mitochondrial membrane polarity in cancer cells enables cationic drugs to accumulate preferentially within mitochondria of them, which may explain the potential selectivity of these drugs for tumour cells.^48^ Fourthly, in the present study, we demonstrate for the first time that pentamidine causes mtDNA reduction in prostate cancer cells. Interestingly, the degrees of mtDNA decrease induced by pentamidine highly correlate with the different sensitivities to pentamidine of prostate cancer cell lines, which indicates that pentamidine may inhibit prostate cancer by targeting mtDNA. However, whether pentamidine does bind to mtDNA of prostate cancer cells requires further verification.

ROS are reactive chemical species containing oxygen such as superoxide anion, hydroxyl radical, hydrogen peroxide or organic peroxides.^49^, ^50^ They are produced intracellularly through multiple mechanisms, and one of the major sources is the mitochondria.^51^, ^52^ The mtDNA reduction and mitochondrial dysfunction can cause a potentially harmful elevation of ROS production,^53^ whereas ROS generation leads to DNA damage, mitochondrial dysfunction and apoptosis.^40^, ^52^ We find that pentamidine increases ROS production, whereas anti‐oxidant NAC decreases ROS and relieves cell apoptosis. In addition, the data demonstrate that NAC alleviates the inhibitory effect of pentamidine on cell proliferation, but the mtDNA reduction induced by pentamidine is not rescued by NAC in prostate cancer cells. Thus, we speculate that the decrease in mtDNA content is not caused by ROS generation. The mechanism responsible for mtDNA reduction induced by pentamidine and the exact sources of elevated ROS remain to be determined in future studies.

In conclusion, we demonstrate that the cationic drug pentamidine can be used as a potent agent to inhibit prostate cancer progression. The suppression of proliferation, migration and invasion in prostate cancer cells is accompanied by a loss of in vivo tumour growth ability. These results highlight the potential of pentamidine as an anti‐cancer agent or a combinational therapy with other approaches for the treatment of prostate cancer.

## CONFLICT OF INTEREST

There is no conflict of interest in this manuscript.

## AUTHOR CONTRIBUTIONS

LL, YL and W‐QG: prepared and revised the manuscript. LL, YL and W‐QG: conceived and designed the experiments. LL, FW and YT: performed the experiments. LL, L‐FL and YL: analysed the data.

## Data Availability

The RNA‐seq data set are shared and can be found in the GEO database with the accession number GSE132693.
