# Supplementary figures and images for: Pentamidine inhibits prostate cancer progression via selectively inducing mitochondrial DNA depletion and dysfunction
Source: Cell Prolif. 2019 Nov 13;53(1):e12718. doi: 10.1111/cpr.12718 (PMC6985668; doi:10.1111/cpr.12718)

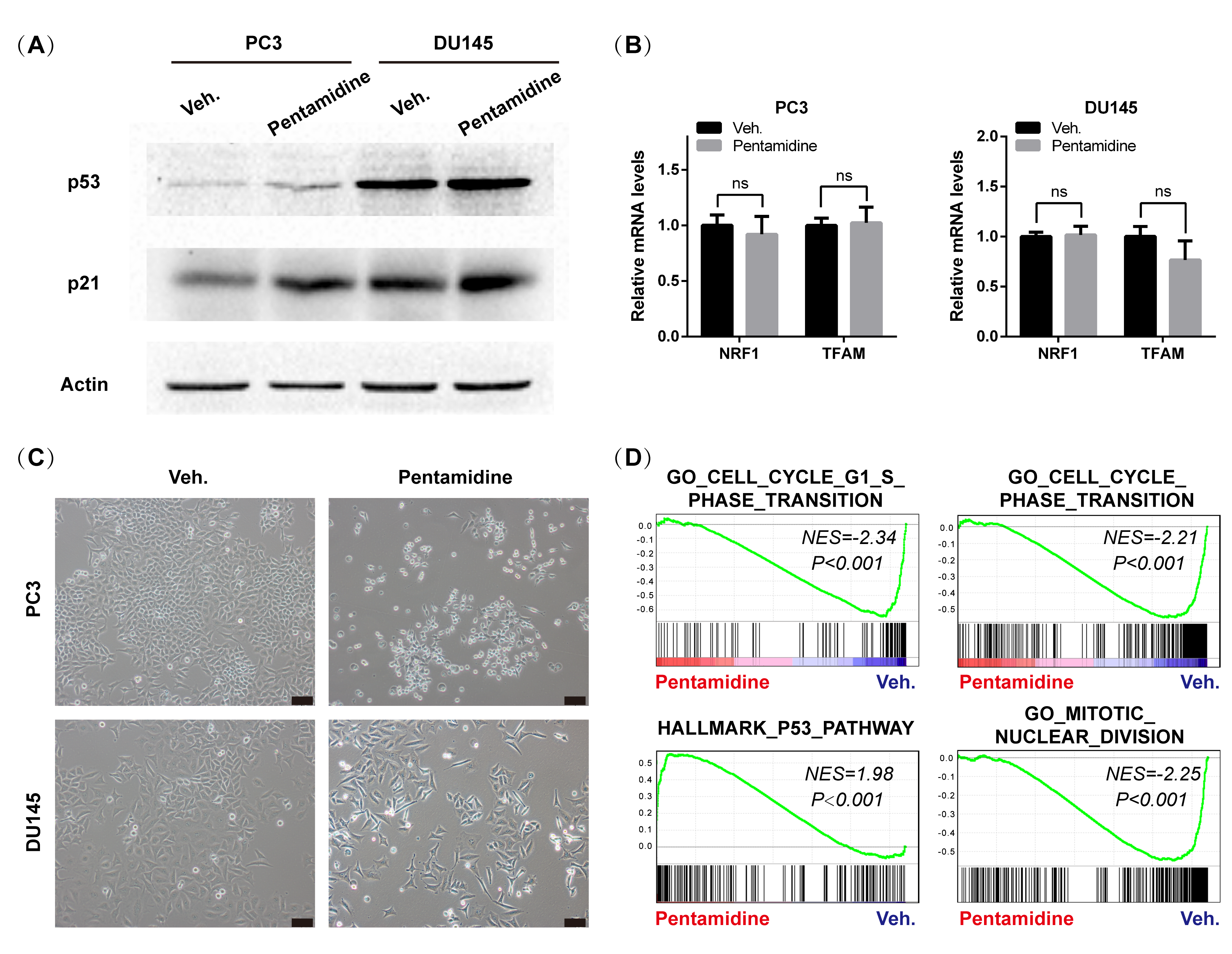

Supplement: Supplementary file 1 [file CPR-53-e12718-s001.tif]

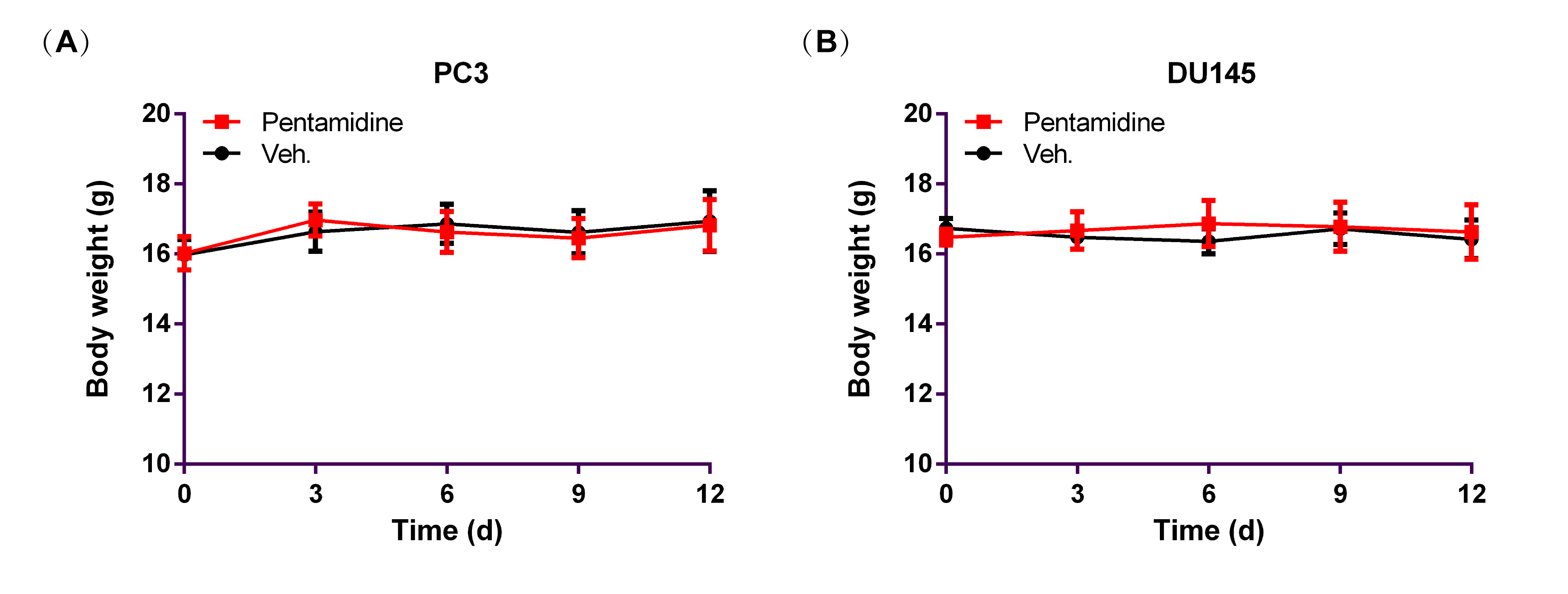

Supplement: Supplementary file 2 [file CPR-53-e12718-s002.tif]
