# Supplementary material for: Pentamidine inhibits prostate cancer progression via selectively inducing mitochondrial DNA depletion and dysfunction
Source: Cell Prolif. 2019 Nov 13;53(1):e12718. doi: 10.1111/cpr.12718 (PMC6985668; doi:10.1111/cpr.12718)
Supplement: Supplementary file 3 [file CPR-53-e12718-s003.doc]

**Supplementary tables**

**Supplementary table 1. Reagents related in this study**

| **Reagents** | **Source** | **Catalog Number** | |
| --- | --- | --- | --- |
| Annexin V-APC | Biolegend (San Diego, CA, USA) | | 640920 |
| Anti-Ki67 antibody | Abcam (Cambridge, MA, USA) | | ab15580 |
| ATP assay kit | Nanjing Jiancheng (Nanjing, China) | | A095 |
| beta Actin Antibody | Proteintech (Wuhan, China) | | 7D2C10 |
| Cell Counting Kit | YEASEN (Shanghai, China) | | 40203ES80 |
| Cell Cycle Staining Kit | MultiSciences (Hangzhou, China) | | CCS01 |
| Cleaved Caspase-3 (Asp175) Antibody | Cell Signaling Technology (Danvers, MA, USA) | | 9661 |
| DAB Peroxidase Substrate Kit | VECTOR (Burlingame, CA, USA) | | SK-4100 |
| Fixing Solution | Servicebio (Wuhan, China) | | G1102 |
| Glucose assay kit | Rsbio (Shanghai, China) | | 361510 |
| HRP-conjugated Affinipure Goat Anti-Mouse IgG | Proteintech (Wuhan, China) | | SA00001-1 |
| HRP-conjugated Affinipure Goat Anti-Rabbit IgG | Proteintech (Wuhan, China) | | SA00001-2 |
| HRP-conjugated Goat Anti-Rabbit IgG | Sangon (Shanghai, China) | | D110073 |
| Instant Immunohistochemistry Kit I | Sangon (Shanghai, China) | | C506333 |
| Matrigel | BD (Franklin Lakes, NJ, USA) | | 356234 |
| Mitochondria Staining Kit (JC-1) | MultiSciences (Hangzhou, China). | | MJ101 |
| MitoTracker Deep Red FM | Invitrogen (Thermo Fisher Scientific, MA, USA) | | M22426 |
| MTCO2 Antibody | Proteintech (Wuhan, China) | | 55070-1-AP |
| P21 Antibody | Proteintech (Wuhan, China) | | 10355-1-AP |
| P53 Antibody | Proteintech (Wuhan, China) | | 10442-1-AP |
| PrimeScript RT Reagent Kit | Takara (Dalian, China) | | RR037A |
| Propidium Iodide | Biolegend (San Diego, CA, USA) | | 421301 |
| Protease inhibitor cocktail | Thermo Scientific (Waltham, MA, USA) | | 87786 |
| QIAamp DNA Micro kit | Qiagen (Hilden, Germany) | | 56304 |
| RIPA Lysis and Extraction Buffer | Thermo Scientific (Waltham, MA, USA) | | 89901 |
| TB Green Premix Ex Taq | Takara (Dalian, China) | | RR420A |
| TRIzol | Invitrogen (Thermo Fisher Scientific, MA, USA) | | 15596018 |
| 2′,7′-Dichlorofluorescein diacetate | Sigma‑Aldrich (Merck, Darmstadt, Germany) | | 35845 |

**Supplementary table 2. The primers used in qPCR assays**

| **Primer** | **Sequences** |
| --- | --- |
| MT-ND1-F | ATGGCCAACCTCCTACTCCT |
| MT-ND1-R | GCGGTGATGTAGAGGGTGAT |
| MT-ND2-F | CATATACCAAATCTCTCCCTC |
| MT-ND2-R | GTGCGAGATAGTAGTAGGGTC |
| MT-ND3-F | TTACGAGTGCGGCTTCGACC |
| MT-ND3-R | ACTCATAGGCCAGACTTAGG |
| MT-ND4-F | CCTGACTCCTACCCCTCACA |
| MT-ND4-R | ATCGGGTGATGATAGCCAAG |
| MT-ND4L-F | TAGTATATCGCTCACACCTC |
| MT-ND4L-R | GTAGTCTAGGCCATATGTG |
| MT-ND5-F | ACATCTGTACCCACGCCTTC |
| MT-ND5-R | TCGATGATGTGGTCTTTGGA |
| MT-ND6-F | GGATCCTCCCGAATCAAC |
| MT-ND6-R | GTAGGATTGGTGCTGTGG |
| MT-CYB-F | TGAAACTTCGGCTCACTCCT |
| MT-CYB-R | AATGTATGGGATGGCGGATA |
| MT-CO1-F | GGCCTGACTGGCATTGTATT |
| MT-CO1-R | TGGCGTAGGTTTGGTCTAGG |
| MT-CO2-F | CAGGAAATAGAAACCGTCTGAACTATCCTG |
| MT-CO2-R | CTGTGGTTTGCTCCACAGATTTCAGTGCAT |
| MT-CO3-F | CCCGCTAAATCCCCTAGAAG |
| MT-CO3-R | GGAAGCCTGTGGCTACAAAA |
| MT-ATP6-F | CACACCTACACCCCTTATCCC |
| MT-ATP6-R | TCATTATGTGTTGTCGTGCAG |
| MT-ATP8-F | ATGGCCCACCATAATTACCC |
| MT-ATP8-R | GCAATGAATGAAGCGAACAG |
| MT-RNR1-F | CCCAAACTGGGATTAGATACCC |
| MT-RNR1-R | GTTTGCTGAAGATGGCGGTA |
| MT-RNR2-F | GCCTGTTTACCAAAAACATCAC |
| MT-RNR2-R | CTCCATAGGGTCTTCTCGTCTT |
| MTTL1-F | TATACCCACACCCACCCAAG |
| MTTL1-R | GCGATTAGAATGGGTACAAT |
| BBC3-F | GACCTCAACGCACAGTACGAG |
| BBC3-R | AGGAGTCCCATGATGAGATTGT |
| TRIB3-F | GCCTTTTTCACTCGGACCCAT |
| TRIB3-R | CAGCGAAGACAAAGCGACAC |
| DDIT3-F | GGAAACAGAGTGGTCATTCCC |
| DDIT3-R | CTGCTTGAGCCGTTCATTCTC |
| HRK-F | TAGGCGACGAGCTGCACCAG |
| HRK-R | CGCACAGCCAAGGCCAGTAG |
| BIRC3-F | TTTCCGTGGCTCTTATTCAAACT |
| BIRC3-R | GCACAGTGGTAGGAACTTCTCAT |
| ASNS-F | GGAAGACAGCCCCGATTTACT |
| ASNS-R | AGCACGAACTGTTGTAATGTCA |
| KLF4-F | CCCACATGAAGCGACTTCCC |
| KLF4-R | CAGGTCCAGGAGATCGTTGAA |
| TFAM-F | ATGGCGTTTCTCCGAAGCAT |
| TFAM-R | TCCGCCCTATAAGCATCTTGA |
| NRF1-F | AGGAACACGGAGTGACCCAA |
| NRF1-R | TATGCTCGGTGTAAGTAGCCA |
| TP53-F | CAGCACATGACGGAGGTTGT |
| TP53-R | TCATCCAAATACTCCACACGC |
| CDKN1A-F | TGTCCGTCAGAACCCATGC |
| CDKN1A-R | AAAGTCGAAGTTCCATCGCTC |
| β-Actin-F | TCCCTGGAGAAGAGCTACG |
| β-Actin-R | GTAGTTTCGTGGATGCCACA |
